# Laboratory-Based SARS-CoV-2 Receptor Binding Domain Serologic Assays Perform with Equivalent Sensitivity and Specificity to Commercial FDA-EUA Approved Tests

**DOI:** 10.3390/v15010106

**Published:** 2022-12-30

**Authors:** Mary Nehring, Sierra Pugh, Tina Dihle, Emily Gallichotte, Terry Nett, Eric Weber, Christie Mayo, Lori Lynn, Greg Ebel, Bailey K. Fosdick, Sue VandeWoude

**Affiliations:** 1 Department of Microbiology, Immunology, and Pathology, Colorado State University, Fort Collins, CO 80523, USA; 2 Department of Statistics, Colorado State University, Fort Collins, CO 80523, USA; 3 Health and Medical Center Laboratory, Colorado State University, Fort Collins, CO 80523, USA; 4 Department of Biomedical Sciences, Colorado State University, Fort Collins, CO 80523, USA; 5 Department of Biostatistics and Informatics, Colorado School of Public Health, Denver, CO 80206, USA

**Keywords:** SARS-CoV-2, serology, surveillance, laboratory diagnostics

## Abstract

During early phases of the SARS-CoV-2 epidemic, many research laboratories repurposed their efforts towards developing diagnostic testing that could aid public health surveillance while commercial and public diagnostic laboratories developed capacity and validated large scale testing methods. Simultaneously, the rush to produce point-of-care and diagnostic facility testing resulted in FDA Emergency Use Authorization with scarce and poorly validated clinical samples. Here, we review serologic test results from 186 serum samples collected in early phases of the pandemic (May 2020) from skilled nursing facilities tested with six laboratory-based and two commercially available assays. Serum neutralization titers were used to set cut-off values using positive to negative ratio (P/N) analysis to account for batch effects. We found that laboratory-based receptor binding domain (RBD) binding assays had equivalent or superior sensitivity and specificity compared to commercially available tests. We also determined seroconversion rate and compared with qPCR outcomes. Our work suggests that research laboratory assays can contribute reliable surveillance information and should be considered important adjuncts to commercial laboratory testing facilities during early phases of disease outbreaks.

## 1. Introduction

Following emergence of SARS-CoV-2 in early 2020, millions of people have been diagnosed with the infection and as of September 2022, more than 6.5 million people have died [1]. Development of specific and sensitive assays have been a critical component in detecting infected patients and contributing to management of the pandemic. While PCR and antigen detection tests have aided identification of infectious individuals, serologic tests have provided evidence of past exposure and determination of the kinetics of humoral immune response to SARS-CoV-2. As of April 2022, 84 Food and Drug Administration-Emergency Use Authorization (FDA-EUA) commercial serologic assays have been approved [2]; including 4 laboratory-based assays at academic institutions or public health laboratories (Emory Medical Laboratory, Mount Sinai Hospital Clinical Laboratory, University of Arizona Genetics Core, Wadsworth Center, New York State Department of Health) [2]. The COVID-19 epidemic catalyzed academic-diagnostic laboratory partnerships, particularly early in the epidemic when testing capacity was lacking [3,4]. Clinical research studies conducted for nondiagnostic purposes do not require FDA Clinical Laboratory Improvement Amendments (CLIA) laboratory validation and verification [5], and thus scientific reports of epidemiology and clinical attributes of COVID-19 conducted using laboratory-based analyses may not be comparable to data reported using FDA approved tests. Different methodologies for determining cut-off values to assess negative, positive, and equivocal tests can also confuse sensitivity and specificity, particularly when a laboratory ‘gold standard’ does not exist.

To assess the usefulness of laboratory-based assays for future epidemics, we therefore conducted a comparison of six laboratory-based COVID-19 serologic assays (ELISA and RadioImmunoAssay using three different SARS-CoV-2 antigens) conducted in two research laboratory settings, and two FDA-EUA approved assays conducted in CLIA laboratory conditions. Samples were collected very early in the pandemic prior to vaccinations or opportunities for multiple exposures and reinfections. One CLIA diagnostic laboratory utilized the first FDA-EUA approved point-of-care test (Cellex qSARS-CoV-2 IgG/IgM Rapid Test, Munich, Germany), while the second laboratory used a commercial antibody test (Abbott Architect SARS-CoV-2 IgG, San Diego, CA, USA). Plaque reduction neutralization tests (PRNT) were performed on a second data set to validate experimental results for spike and RBD ELISAs while the CLIA certified Abbot was used for nucleocapsid ELISA [6]. We screened for the presence of SARS-CoV-2 IgG antibodies in 186 staff working at skilled nursing facilities from sites across Colorado collected in May 2020 [7]. A subset of participants was also tested for SARS-CoV-2 viral RNA in nasopharangeal swabs by a laboratory-based qPCR, confirmed by CLIA laboratory testing on a weekly basis for 1–6 weeks prior to blood collection [6]. We further considered a variety of cut-off methods using PRNT as a gold standard and selected P/N ratio analysis to account for the effect of running samples in batches on different plates. This unique sample collection illustrated the potential for laboratory-based assays to aid in pathogen surveillance during early epidemics with accuracy equivalent to early commercial products, when commercial products were still relatively scarce.

## 2. Materials and Methods

### 2.1. Sample Collection

Participants consented to participate in the study, which was reviewed and approved by Colorado State University IRB (CSU protocol 20-10057H). Nasopharyngeal swabs were collected and transferred to viral transport media. RNA was extracted from the sample and tested for viral RNA using CDC primer/probes using quantitative real-time PCR as previously described [7]. Serum was collected from 186 staff at six skilled nursing facilities for retrospective point-in-time blood analyses [7]. Serum was stored at −80 °C and heat inactivated at 56 °C for 30 min prior to testing.

### 2.2. Serologic Assays

#### 2.2.1. Laboratory-Based ELISA

RBD and spike ELISAs were modified from Amanat et al. and conducted as previously described [6,8]. ELISA plates were coated either with RBD (Sino Biological, Wayne, PA, USA), nucleocapsid (Gift from B. Geiss Lab) or spike protein (Sino Biological, Wayne, PA, USA). Samples and controls were added after 1 h of blocking (1X PBS, 3% milk powder, 0.1% tween). Positive controls included convalescent patient serum (gift of R. Goodrich) and monoclonal antibody CR3022 (Absolute Antibody, Boston, MA, USA). Negative control serum was charcoal inactivated pooled human serum collected in 2015 (Jackson Immuno Research, West Grove, PA, USA). Secondary antibody was added followed by development and reading via spectrophotometer (Thermo Fisher, Dallas, TX, USA).

#### 2.2.2. Statistical Analysis

**Figure 1:**
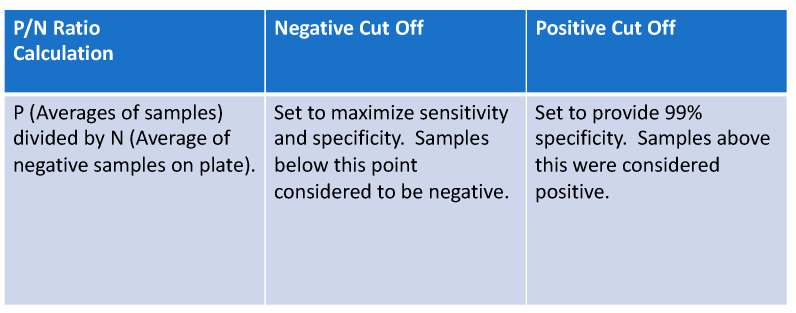
P/N ratio formula was used to set cut-off points for positive, equivocal, and negative results in laboratory-based assays.

**Figure 2:**
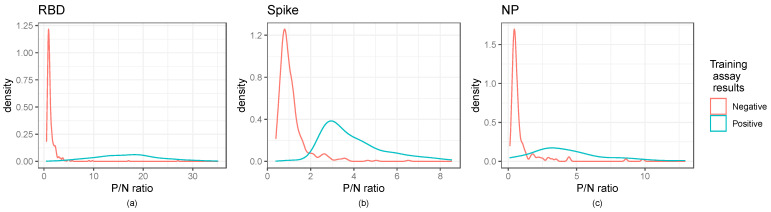
Distribution of P/N ratios illustrate cut-off values for ELISA assays: Kernel density estimates of the P/N ratios for the ELISA assays. Panels (**a**–**c**) illustrate RBD, Spike, and NP ratios, respectively. The RBD and spike panels show the secondary training data, while the nucleocapsid panel shows the dataset of interest. Training assay data results are those of the neutralization assay for RBD and spike and the Abbott results for the nucleocapsid panel.

The presence/absence of antibodies based on the ELISA results were determined by creating cut-off values for each assay. To account for the batch effects resulting from running the samples on different plates, we calculated P/N ratios as the ratio of the average sample OD values and the average negative control values run on the same plate as the sample, similar to Zhang et al., 2013 [9] (see Figure 1). We used an additional data set of 690 samples from two long term care facilities to set the P/N ratio cut-offs for spike and RBD to avoid overfitting (Figure 2) [6]. Within this second data set, neutralization assay results were considered as “truth” for spike and RBD ELISAs. We substituted Abbott test results for our dataset of interest as “truth” for the nucleocapsid ELISA.

The “lower” cut-off for each assay was set at the value maximizing the sum of sensitivity and specificity (equivalent to maximizing Youden’s index) [10]. For the “upper” cut-off values, we followed CDC recommendations for specificity and sensitivity analysis [11], setting the upper cut-offs at the 0.99 quantiles of the negative controls for each assay, ensuring an empirical 99% specificity. We followed this nonparametric approach to estimating the 0.99 quantile rather than a parametric approach to account for the non-normal distribution of the negative controls (Table S1). We then defined any observation with a P/N ratio less than both cut-offs as negative, any observation between the two cut-offs as equivocal, and any observation above both cut-offs as positive. Once cut-off values were established for ELISA and RIA analyses, assessment of positive, negative, and equivocal samples was made. These results were compared to qPCR results, RIA, and results from the two commercial assays (full dataset and results for each sample can be found in Supplemental Dataset S1).

#### 2.2.3. Laboratory-Based RadioImmunoAssay

Radioactive RBD was prepared by incubating 5 ug recombinant RBD with 1 mCi of Na-^125^I in the presence of chloramine T for 2 min at room temperature [12]. Sodium metabisulfite was used to terminate the reaction and the contents of the reaction tube were transferred to a 10 mL Sephadex G-25 column. One ml fractions were collected in PBS (pH 7.0) containing 0.1% gelatin (PBS-gel). The ^125^I-RBD eluted in fractions 4–6 and free Na-^125^I eluted in fractions 10–13. Specific activity of the ^125^I-RBD was calculated by measuring the amount of ^125^I incorporated into the RBD (13.6%). Thus, the specific activity was calculated to be 27.2 uCi/ug RBD.

For analysis, duplicate aliquots of 25 ul patient serum was incubated with ~25,000 cpm ^125^I-RBD in 100 ul of phosphate-buffered saline (0.01 M, pH 7.0) containing 0.1% gelatin for 24 h at 4 °C. At that time, 10 ul Protein G PLUS-Agarose beads (Santa Cruz Biotechnology, Dallas, TX, United States) were added to each tube. Tubes were put on a Fischer Clinical Rotator at 180 rpm and incubation was continued overnight at 4 °C. After this incubation, 3 mL of cold PBS containing 10 ug pituitary membranes were added to each tube. The membranes provided a matrix to trap the beads so that the samples could be centrifuged and the supernatant decanted. Immediately after the addition of the cold PBS, samples were centrifuged at 2800× *g* for 15 min. The supernatant was decanted and the radioactivity in the precipitate was quantified using an automatic gamma spectrometer.

Sensitivity of the assay was determined by calculating the 95% confidence limit of radioactivity precipitated in a known negative (pre-pandemic) sample of serum (nonspecific binding, NSB). Any sample whose radioactive counts exceeded the NSB by at least 2 times the 95% confidence limit were considered positive for antibody to SARS-CoV-2. If the radioactive counts were 1–2 times the 95% confidence limit for the NSB, they were considered equivocal. Those samples whose counts did not exceed the NSB by more than 1 times the NSB were considered negative. A similar analysis was performed on each sample using ^125^I-S or ^125^I-NP prepared in the same manner as the ^125^I-RBD.

#### 2.2.4. Commercial Point of Care Assay

The first FDA Emergency Use Authorized (EUA) serologic point-of-care assay was offered for sale by Cellex (Morrisville, NC, United States, State [13]. Cellex kits (Cat. No:5515C025, 5515C050 and 5515C100) were run following manufacturer’s instructions in a CLIA certified lab to test for IgG and/or IgM reactivity against SARS-CoV-2 recombinant spike and nucleocapsid proteins [13]. Frozen serum samples were thawed at room temperature and added to the testing cassette. Ten microliters of each sample was added to the testing well followed by 2 drops of sample diluent [13]. Tests were incubated for 10–15 min. Positive or negative was visually determined by comparing the developed red line next to either IgG or IgM indicators.

#### 2.2.5. Commercial Laboratory-Based Assay

Samples tested via Abbott Architect SARS-CoV-2 IgG were tested at the National Jewish Health Advanced Diagnostic Laboratory. Samples were tested for IgG/IgM antibodies against the recombinant SARS-CoV-2 nucleocapsid protein [14].

#### 2.2.6. Laboratory-Based Functional Virus-Neutralization Assay

Plaque reduction neutralization test (PRNT) was run on 45 of the 186 samples. PRNT was run on a second data set (690 samples) from two long term care facilities and used to set the cut point for the RBD and spike ELISAs [6]. PRNT serum samples were serially diluted in DMEM containing 1% FBS and mixed with SARS-CoV-2 virus (2019-nCoV/USA-WA1/2020 strain) in a BSL3 facility. Virus-antibody mixes were added to Vero cells, overlaid with a tragacanth medium, and incubated for 2 days. Plates were stained with crystal violet to identify viral plaques. Samples with 50% neutralization titers less than <1:20 were considered negative and plotted at half the limit of detection [10].

## 3. Results

**Figure 3:**
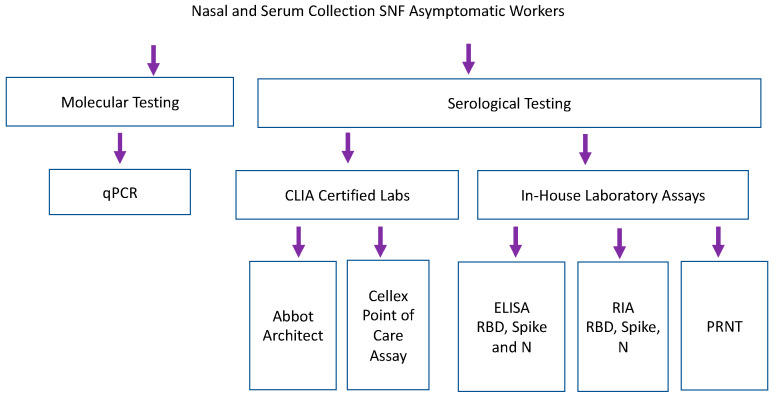
Serum samples collected retrospectively during longitudinal SARS-CoV-2 PCR surveillance were tested by commercial and laboratory serologic assays. Laboratory-based Enzyme Linked Immunosorbent Assay (ELISA) and RadioImmunoAssays (RIA) assessed binding of IgG against recombinant RBD and Spike (Sino Biologics) and nucleocapsid antigen (manufactured in house, courtesy of *B. Geiss*).

Samples collected from nursing home facilities were tested by qPCR, CLIA certified serological testing, and in-house lab assays including RBD, spike and nucleocapsid ELISAs and RIA (Figure 3). Staff participated in weekly qPCR surveillance testing at 6 different skilled nursing facilities [7]. Serum was collected from a single time point, 1–6 weeks after surveillance testing began [7]. 186 samples were analyzed by eight antibody binding assays (2 CLIA laboratory assays, 3 laboratory-based ELISAs and 3 laboratory-based RIA). Forty-five samples were run on functional PRNT. Abbott Architect assessed IgG and IgM versus SARS-CoV-2 nucleocapsid antigen [14]. Cellex point of care assay assessed IgG and IgM against nucleocapsid and spike antigens [13]. Because samples were taken in May 2020, none of the study participants were vaccinated against COVID-19, and a COVID-19 infection more than two months prior to the study is quite unlikely given the United States lockdown, which began in March of 2020.

As shown in Figure 2, kernel density estimates of the P/N ratios for the ELISA indicated that negative samples had a right skewed, non-normal distribution, highlighting the need for cut-off selection methods that do not rely on assuming a normal distribution. Normal distribution-based cut-off methods for obtaining a 0.99 specificity were shown to have lower than the anticipated specificity of 0.99 (Table S2). Figure 2 further indicates the distinction between positive and negative controls.

**Figure 4:**
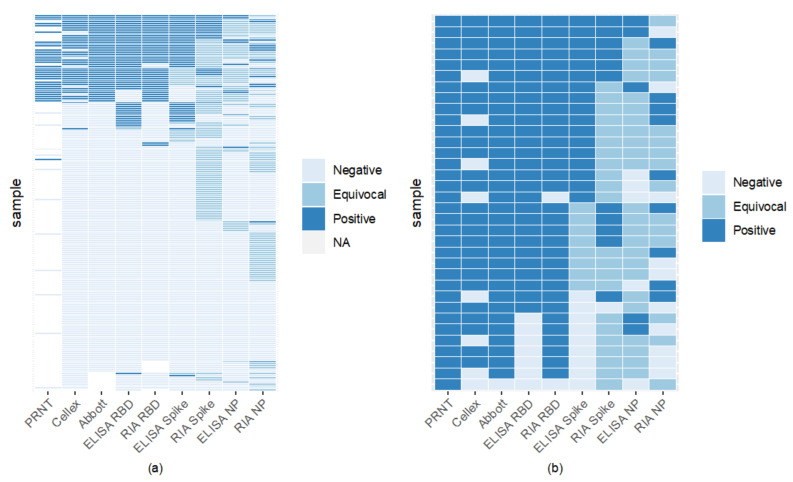
Inter-assay comparisons reveal high RBD binding assay sensitivity and specificity. Each horizontal line indicates an individual sample tested by one of 8 binding assay methods. ELISA values below the negative cut-off determined by P/N were deemed negative; those above the positive cut-off were considered positive. Values between upper and lower cut-offs were equivocal. 66% (122/186) of samples were seronegative by all serological assays (light blue lines). Panel (**a**) includes samples tested on PRNT and all 8 binding assays. Panel (**b**) compares PRNT positive samples (gold standard positive) to the 8 other serological assays.

Comparison of assays found RBD to have high binding, with RIA and ELISA RBD to be the most agreeable (Figure 4). Sixty-six percent of samples were seronegative across all assays (Figure 4). RBD ELISA and RIA were the most aligned with the FDA-EUA assays, having a minimum of 87% agreement, while RBD and spike ELISA had the highest concordance among the laboratory-based assays of 91% (Figure 4, Table S3). Nucleocapsid ELISA and RIA showed the lowest concordance with CLIA laboratory assays, with most of their agreement coming from the samples considered negative by both assays.

**Figure 5:**
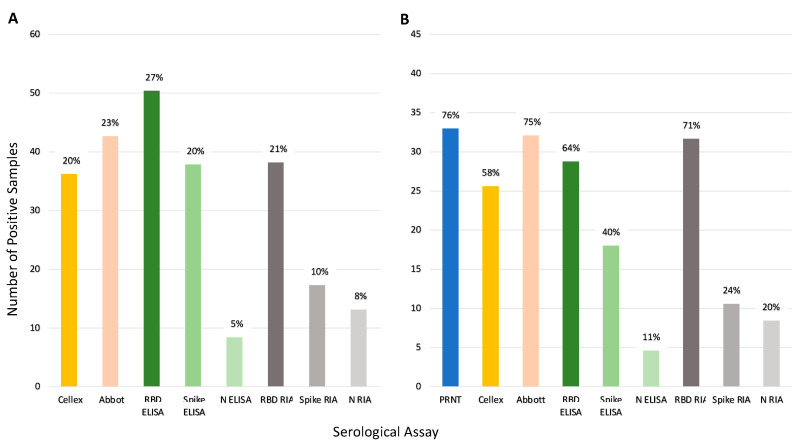
Comparison of seroprevalence predicted by each serologic assay tested. Percentages represent positive samples by each assay. Panel (**A**) shows total percentage of positive samples (*n* = 186). Panel (**B**) shows the percentage of positive samples for the samples run on PRNT (*n* = 45). Note: Of the 45 samples only 44 samples were run on Abbot. One was not classified resulting in 33 out of 44 samples being positive (75%).

Figure 5 illustrates the seroprevalence estimate for each assay based on all the samples (Panel A) and for the subset of 45 samples run on PRNT (Panel B). Abbott and RBD RIA estimated comparable seroprevalence for all samples (23% and 21%, respectively), and Panel B shows these assays estimated comparable seroprevalence with the PRNT results as well. Inter-assay agreement for Abbot and RBD RIA was high (98% samples had concordant results, see Table S3). Both Cellex and the spike ELISA assays estimated a seroprevalence of 20% across all samples and estimated a lower seroprevalence of the PRNT samples, 58% and 40%, respectively, compared to the 76% of PRNT. The spike ELISA estimates a lower seroprevalence than PRNT likely due to a lowered sensitivity; only 47% of the positive controls in the validation dataset were correctly identified by this assay (Table S1), and only half of the samples that tested positive with PRNT tested positive with spike ELISA (Figure 4, Panel b). Nucleocapsid ELISA and RIA along with spike RIA were insensitive with particularly low seroprevalence estimates.

**Figure 6:**
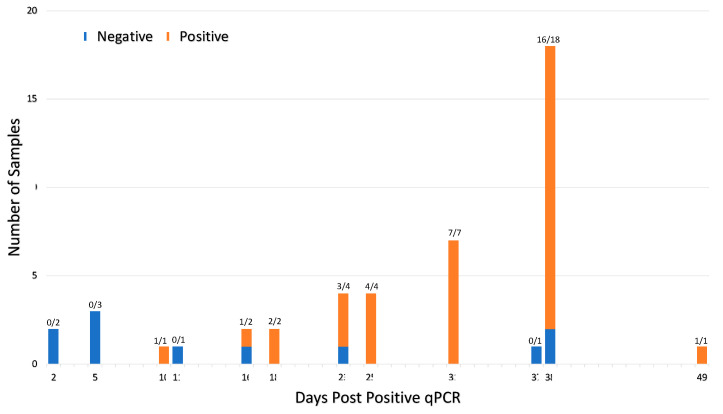
Seroconversion occurs within 10–49 days of viral infection for 46 qPCR positive participants. Forty-six participants tested qPCR positive prior to serum collection. The ratio above each bar shows number of seropositive cases per the total number of samples tested at each timepoint. Samples were deemed seropositive if it was positive on at least one of the eight serological assays. The interval between positive qPCR and sample collection varied from 2–49 days due to the retrospective nature of the study. Seroconversion was detected in 76% (35 of 46) qPCR positive patients.

Comparing serological assays to qPCR, of the 46 participants that tested positive via qPCR, 35 samples were positive for viral nucleic acid antigens and antibodies as determined by at least one assay (Table 1). Seroconversion (as measured by any one of the eight serological assays) occurred within 10–49 days post infection in 76% of patients (Figure 6). Table 1 shows Abbott and RIA RBD had the highest number of qPCR positive samples, testing positive with 73% and 70%, respectively. RIA Spike, ELISA NP, and RIA NP had particularly low agreement with less than 25% testing positive. Of the qPCR negative samples, all but ELISA RBD and ELISA spike had less than 10% of the samples test positive. Given the period, false positive ELISA RBD and ELISA spike seems most likely due to a lack of sensitivity versus finding a prior infection or a false negative qPCR (these samples were tested before the variant Omicron made qPCR testing less reliable).

## 4. Discussion

Because of the urgent need for access to COVID-19 antibody tests, the FDA granted EUA authority for available diagnostics early in the SARS-CoV-2 pandemic using relatively limited quality assurance data, while monitoring test performance to withdraw approvals if necessary. As a result, the accuracy of some tests available early in the pandemic was not optimal [16]. As of March 2021, 259 manufacturers of SARS-CoV-2 serological tests had been removed from the FDA’s list either due to low performance, lack of EUA application, or withdrawal from manufacture [17]. The high degree of laboratory technical capacity in academic research laboratories represents a resource for filling in gaps in available testing; thus, we sought to evaluate performance of two CLIA-certified antibody detection methods compared to laboratory-based assays in a population with high COVID-19 disease incidence.

Of the 84 FDA-EUA approved serological assays, 58 evaluate antibodies against spike protein; 54 of these detect IgG and 29 assess both IgG and IgM [2]. RBD IgG titers highly correlate with neutralizing antibodies in hospitalized patients [18] while RBD IgA have higher sensitivity and specificity although with lower optical density (OD) values compared to IgG and IgM [19]. Evaluation of commercial ELISAs, including Abbott Architect SARS-CoV-2 IgG have shown sensitivity as high as 100% 11–17 days post onset of symptoms [20,21] and specificity ranging from 95.1–99.9% [21,22].

Our results illustrate that two RBD laboratory-based antibody (ELISA and RIA) assays evaluating seroantibodies were comparable (Figure 4). One commercially produced assay (Cellex) and spike ELISA had comparable positive results (37 versus 38); however, which samples they identified as positive differed (Figure 4). Nucleocapsid assays including Abbott, ELISA, and RIA had the most discordance between assays with the laboratory assay finding few (9–14) positive cases, but the commercially available Abbott having higher sensitivity with 43 positives (Figure 5, Table S4). The commercially available Abbott test performed closely compared to a subset of 45 samples tested via PRNT (Figure 5). Variation between laboratory and commercial nucleocapsid analysis may in part be due to Abbot testing for both IgG and IgM, while laboratory ELISA and RIA only analyzed IgG. Cellex comparability analyzed both IgG and IgM against spike and nucleocapsid, but still had lower sensitivity. Our results confirm that detection of IgG against RBD antigens offers a sensitive and specific alternative to more cumbersome and time-consuming virus neutralization assays, whereas serologic assays based upon antibodies against nucleocapsid are less sensitive. Analysis of nucleocapsid antibodies can, however, assist in distinguishing individuals that have experienced COVID-19 infection versus those vaccinated with mRNA RBD preparations [23]. In lab, testing focused on spike and RBD recombinant proteins yielding higher sensitivity.

Numerous methods have been proposed in the literature for setting cut-off values for test assays [9,24,25]. Many are based on the raw OD values and rely on a cut-off based on assuming the negative samples follow a normal distribution. Such methods attempt to guarantee a desired specificity for the test; however, their actual specificity can differ greatly from the desired level if the negative samples are not normally distributed as was the case for our sample. Further, many methods do not account for batch effects, which we correct for using the P/N method. Setting a P/N ratio can additionally allow for the use of ELISAs when the gold standard of testing may not be readily available.

Comparison of qPCR and serologic data in our pre-vaccination cohort illustrates multiple individuals without reported SARS-CoV-2 infection had significant antibody responses, indicating previously infected individuals who had not been tested via qPCR because of the lack of test availability during early phases of the epidemic (Table 1). Abbott BinaxNOW antibody detection identified seropositive patients with qPCR Ct values 25–30 [26,27], but not in patients where viral loads were lower than 4.04–8.06 copies/swab [27]. In one population, this corresponds to missing 40% of qPCR positive in-hospital patients [27]. Assessment of seroconversion in comparison to qPCR testing identified significant individual variation in time to seroconversion (Figure 6) and provided a characterized bioarchive for future serologic test development.

During early phases of COVID-19, many research laboratories repurposed their expertise to contribute to information about SARS-CoV-2, including assessment of genomic content [28], serum antibody profiles [8], and qPCR [7]. Our work supports a pre-clinical addition to the diagnostic pipeline process that should be considered for adoption for early phases of infectious disease outbreaks in humans, animals, or plants while commercial and public diagnostic facilities gear up clinical diagnostic capabilities.

## Figures and Tables

**Table 1:** Serologic assessments augmented qPCR findings in describing individual SARS-CoV-2 infection status. The proportion of positive antibody test results compared to the qPCR result for each test. “All assay” shows the number of samples with a positive result from any of the eight antibody tests. Serologic screening identified 29 individuals that were qPCR negative that had previously been exposed to virus, and 11 individuals were positive for qPCR but seronegative, presumably tested prior to seroconversion. Positive and negative predictive for the positive result from any test was 55% (35/64) and 90% (111/122) [15].

All Serological Assays Compared to qPCR										
qPCR	Antibody									
			Positive				Negative			
	Positive		35				11			
	Negative		29				111			
**Individual Assays Compared to qPCR**										
		Cellex	Abbott	ELISA RBD	RIA RBD	ELISA Spike	RIA Spike	ELISA NP	RIA NP	Any test
qPCR	Positive	0.57 (26/46)	0.73 (33/45)	0.63 (29/46)	0.70 (32/46)	0.39 (18/46)	0.24 (11/46)	0.11 (5/46)	0.20 (9/46)	0.76 (35/45)
	Negative	0.08 (11/139)	0.08 (10/132)	0.16 (22/140)	0.08 (11/133)	0.14 (20/140)	0.05 (7/140)	0.03 (4/140)	0.04 (5/140)	0.21 (29/140)

## Data Availability

Data for this paper is supported in supplementary tables and datasets.

## Supplementary Materials

The following supporting information can be downloaded at: https://www.mdpi.com/article/10.3390/v15010106/s1, Table S1: Positive and Negative Cut-Offs for the ELISA Assays in Terms of P/N ratios; Table S2: Training Dataset for RBD and Spike ELISAs; Table S3: Agreement of Results Between Assays; Table S4: Sample Classification by Assay; Dataset S1: Results of COVID Assays for Each Sample.
