# Supplementary material for: Laboratory-Based SARS-CoV-2 Receptor Binding Domain Serologic Assays Perform with Equivalent Sensitivity and Specificity to Commercial FDA-EUA Approved Tests
Source: Viruses. 2022 Dec 30;15(1):106. doi: 10.3390/v15010106 (PMC9860642; doi:10.3390/v15010106)
Supplement: Supplementary file 1 [file viruses-15-00106-s001.zip › viruses-2127741-supplementary.v1.pdf]

Table S1: Positive and negative cut offs for the ELISA assays in terms of P/N ratios. Sensitivity and specificity are estimated using neutralization assay results from the secondary verification dataset of 690 samples from two long term care facilities as the "truth" for RBD and spike ELISA, and using Abbott results from the dataset of interest as the "truth" for the NP ELISA.

		Cut-off	Sensitivity	Specificity
**RBD ELISA**	Positive cut off	5.42	0.97	0.99
	Negative cut off	4.42	0.98	0.98
**Spike ELISA**	Positive cut off	3.58	0.47	0.99
	Negative cut off	2.19	0.99	0.94
**NP ELISA**	Positive cut off	7.24	0.16	0.99
	Negative cut off	2.00	0.88	0.91

Table S2: **Training Dataset for RBD and Spike ELISAs.** The empirical specificity of the training dataset for the ELISA RBD and ELISA Spike assays under various cut off methods. Negative controls are identified by the neutralization assay results

Test	Cut off method	Specificity	
ELISA RBD	Youden	0.98	(437/445)
	Nonparametric	0.99	(440/445)
	Parametric	0.99	(440/445)
	Transformed	0.99	(441/445)
ELISA Spike	Youden	0.94	(417/445)
	Nonparametric	0.99	(440/445)
	Parametric	0.96	(426/445)
	Transformed	0.98	(435/445)

The cutoffs are calculated as follows:

- **Youden:** maximizes the sum of the sensitivity and specificity
- **Nonparametric:** calculates the empirical 99th percentile of the negative controls
- **Parametric:** calculates the 99th percentile of a normal distribution with a mean and standard deviation equal to the sample mean and standard deviation of the negative controls
- **Transformed:** The Box Cox transformation was used to transform the negative control P/N ratios to be approximately normal. The parametric method was then applied to the transformed P/N ratios.

Table S3: **Agreement of Results Between Assays**. Proportion of samples with agreeing results for each combination of test assays in the dataset of interest, treating equivocal results and negative results as equal.

	Cellex	Abbot	RBD ELISA	Spike ELISA	NP ELISA	RBD RIA	Spike RIA	NP RIA
Cellex	-	0.94	0.87	0.84	0.83	0.93	0.86	0.83
Abbot	0.94	-	0.89	0.84	0.79	0.98	0.85	0.81
RBD ELISA	0.87	0.89	-	0.91	0.74	0.87	0.82	0.78
Spike ELISA	0.84	0.84	0.91	-	0.81	0.82	0.84	0.81
NP ELISA	0.83	0.79	0.74	0.81	-	0.78	0.88	0.89
RBD RIA	0.93	0.98	0.87	0.82	0.78	-	0.85	0.82
Spike RIA	0.86	0.85	0.82	0.84	0.88	0.85	-	0.87
NP RIA	0.83	0.81	0.78	0.81	0.89	0.82	0.87	-

Table S4: **Sample Classification by Assay**. Number of samples that were classified as negative, equivocal, positive, or not classified for each assay.

Test	Negative	Equivocal	Positive	Not classified
Cellex	148	0	37	1
Abbot	134	0	43	9
RBD ELISA	134	1	51	0
Spike ELISA	131	17	38	0
NP ELISA	135	42	9	0
RBD RIA	136	0	43	7
Spike RIA	90	78	18	0
NP RIA	103	69	14	0
